# Development of Ecofriendly Snail Shell Particulate-Reinforced Recycled Waste Plastic Composites for Automobile Application

**DOI:** 10.1155/2020/7462758

**Published:** 2020-07-17

**Authors:** I. O. Oladele, A. A. Adediran, A. D. Akinwekomi, M. H. Adegun, O. O. Olumakinde, O. O. Daramola

**Affiliations:** ^1^Department of Metallurgical and Materials Engineering, Federal University of Technology Akure, PMB, 704, Akure, Ondo State, Nigeria; ^2^Department of Mechanical Engineering, Landmark University, PMB 1001, Omu-Aran, Kwara State, Nigeria; ^3^Department of Mechanical and Aerospace Engineering, The Hong Kong University of Science and Technology, Hong Kong, Hong Kong

## Abstract

The increase in demand for thermoplastics as a light-weight material for automobile application and other commercial purposes prompts more research into the available polymer resources. In this research, the possibility of enhancing the performance of recycled waste plastics (RWP) as polymer-based composites was examined. Particulate snail shell was obtained by grounding and sieving snail shells to obtain 53–63 *μ*m passing which was used as reinforcement in the recycled waste plastics. The composites were developed by adding varying proportions of the snail shell particulate (SSP) to RWP using a randomly dispersed process in a hot compression moulding machine maintained at 190°C for 7 min. Selected properties of SSP-reinforced RWP composites were examined. The results showed an appreciable enhancement in the properties of composites developed compared to an unreinforced RWP matrix that serves as control. The ultimate tensile strength was enhanced by about 64%, while Young's Modulus and impact strength were enhanced by 37% and 29%, respectively. Wear and water repellant potentials were highly enhanced with the addition of 15 wt% of SSP with values of about 52% and 91%, respectively. This revealed that high content of the SSP contributes to the improvement of the strain-hardening potentials of the developed composites. The results showed that this composite material can be suitably adapted for use in the interior of automobiles as door sills or the floor panel.

## 1. Introduction

Polymer matrix composites (PMCs) are made by incorporating reinforcement into the matrix of thermoplastic and thermosetting materials. Their characteristics such as lightweight, high stiffness, high strength, good corrosion resistance, lesser environmental degradation, excellent thermal insulation, good acoustic damping, excellent design flexibility, and nonmagnetic properties have broadened the spectrum of their applications [1, 2]. They are currently replacing conventional materials such as metal, ceramics, and wood in diverse applications that require light weight materials [3–6]. However, one of its major shortcomings is the disposal problem because it takes a longer time for polymeric materials to degrade. Thus, their perpetual existence often causes environmental pollution. Even though the economical means of disposing these polymeric wastes is to recycle and reuse them for the development of secondary materials, this has not been highly promoted by researchers. Various studies have been carried out on the development of biodegradable plastic-based composites by using natural fibres as reinforcement [7, 8]; however, damaged polymeric materials are often incinerated, and the burning process causes release of toxic gases to the environment [9, 10]. Notwithstanding, the merits in polymers coupled with incessant increase in population definitely increase their demand for various applications. Thus, the emergence of recycling processes with the introduction of additives to help replenish the physical and mechanical properties of the recycled polymer is a good initiative. Grigore [11] accentuated that recycling helps to overcome the environmental problems associated with incineration and ensure the recovery of materials and energy. Agunsoye and Aigbodion [12] prepared bagasse-filled recycled polyethylene biocomposites and reported improvement in the mechanical properties. Also, research studies aimed at improving the mechanical properties of PMCs have investigated the feasibility of readily available fillers such as fly ash, mica, snail shell, coconut shell, calcite, rice husk, and periwinkle shell [13, 14]. Oladele et al. reported a significant enhancement in wear and mechanical properties of epoxy biocomposite samples reinforced with a smaller particle size of African land snail shell with lower filler loading [15]. Similarly, Adeyanju et al. developed polyester composites using snail shell particulate. They reported improvement in the properties of composition with 5–20% snail shell particulate [16]. Thus, the utilization of snail shell particles as reinforcement provides an environmental friendly means of utilizing this animal waste. Snail shell particulate is a potential reinforcement due to the presence of a hard and rigid CaCO_3_ phase [17]. In this study, the viability and environmental feasibility of utilizing snail shell particles as fillers in recycled waste plastic for interior automobile application were investigated. This became necessary because of regulations in the developed nations for the inclusion of high proportion of biodegradable materials in automobiles [18–22]. This work seeks to promote the use of secondary materials rather than creating new ones for the growing global demands for new materials. There is need to intensify efforts towards identifying areas of applications suitable with the potentials imbedded in derived materials. The waste plastic was from polypropylene because it is one of the mostly used polymers for the automobile interior. Polypropylene (PP) is a crystalline thermoplastic polymer, and due to its excellent properties such as high heat distortion temperature, transparency, flame resistance, and dimensional stability, it is extensively used for automobile, industrial, and household purposes [19, 23–25].

## 2. Materials and Methods

**Figure 1:**
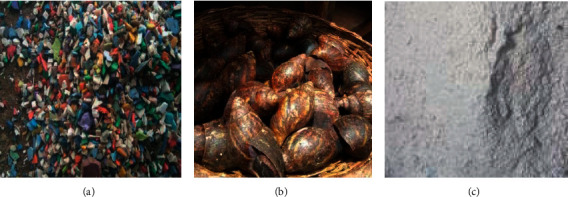
(a) Waste plastics, (b) snail shell, and (c) snail shell particle.

Damaged plastic chairs and tables were obtained from Black Horse Plastics Industry in Ibadan, Nigeria. Snail shells were sourced from a farm settlement in Ibadan, Oyo State, Nigeria. Dioctyl phthalate and zinc stearate which were used as the plasticizer and stabilizer were purchased from a commercial supplier in Edo State, Nigeria. Figures 1(a)–1(c) show the plastic waste, the snail shell, and the pulverized snail shell used in this study.

### 2.1. Processing of Waste Plastics and Snail Shell

The waste plastics and snail shells were washed with tap water to remove the adhered dirt and sun dried for 3 days. The waste plastics were shredded with a shredding machine to obtain granules, while the snail shells were crushed manually using a grinding stone and, later, pulverized using a pulverizing machine. A vibratory sieve shaker (16155 model) was used to filter the fine particles into a particle size range of 53–63 *μ*m.

### 2.2. Development of Recycled PP Composites

**Figure 2:**
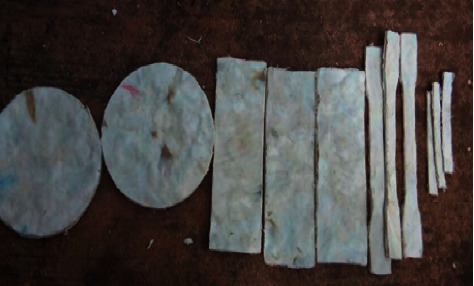
Developed composite samples.

The PP composite samples were produced using a hot compression moulding machine maintained at 190 C and for 7 min. The PP granules were mixed with a varying content of snail shell particulate from 3–15 wt%. Development of polymer composites from solid materials involves two stages: compounding and fabrication of the final sample. Compounding of samples was carried out to ensure even distribution of the reinforcement in the matrix and a proper blend. This is necessary due to variation in densities of the constituents to avoid segregation during fabrication. This initial stage was followed by reproduction of the sample but with the addition of dioctyl phthalate and zinc stearate using the same working parameters. The ratio of dioctyl phthalate to zinc stearate that were used as the plasticizer and stabilizer, respectively, was 3 : 1. The particles for each composition were thoroughly mixed manually with PP for compounding. During compounding, the samples were compacted in a rectangular mould with a laboratory compression moulding machine and also grinded with a laboratory polymer grinding machine. After compounding, the samples were filled into different moulds for the different tests to be carried out, respectively, and were placed inside the compression moulding machine operated at 190°C and 4 MPa for 7 minutes to produce each sample. The samples were air cooled at ambient temperature for 20 ± 3 minutes before being removed from the moulds. A similar production procedure was carried out for the control sample (100% RWP). Three samples were produced for each test composition.  Table 1 shows composition for the developed recycled polypropylene-based composites, while Figure 2 shows the representative samples for the selected test such as wear, water absorption, and mechanical property.

### 2.3. Mechanical Property Testing

Tensile, flexural, and impact tests were used to determine the mechanical properties of the SSP-reinforced RPP composites. The tensile tests were performed using an Instron machine, Instron Computerised Mechanical Universal Testing Machine, Series 3369, with a load cell capacity of 50 kN in accordance with ASTM D3038M-08 standards [26]. Dumb-bell-shaped samples produced during the moulding process were used for the tensile tests. Three-point flexural tests were used to determine the flexural strength of the recycled composites. The flexural tests were performed in accordance with the ASTM D7264M-07 standard [27] using a tensiometric universal testing machine with a crosshead speed of 0.3 mm/min and at a specific strain rate of 10^−3^/s. Notched izod samples were used to carry out the impact test in accordance with the ASTM D256-10 standard [28] using a Housefield balanced impact testing machine. The notched samples were placed in a cantilever position, with the notched surface directly opposite to the swinging pendulum. The pendulum of the testing machine was swung freely through 180° to fracture the samples. To ensure accuracy and reliability of the test results, three repeatability tests were performed for each composition.

### 2.4. Wear Test

The wear-resistance test of the recycled PP composites was carried out on the Taber Abrasers, Model ISE-AO16. The samples were mounted on a turn table platform that rotates at a fixed speed in accordance with [29]. The sample was weighed using an analytical weighing balance for the initial weight of the sample, after which it was fixed on the turn table. The turn table platform was made to rotate at 1000 rpm for 5 hours, and then, the samples were removed and weighed to obtain the final weight of the sample. The differences in the initial and final values of the samples were used to determine wear behavior.

### 2.5. Water Absorption

A water absorption test was carried by immersing the recycled PP composites into 250 cm^3^ of distilled water for 7 days. The percentage difference between the initial and the final weight of each of the sample was computed. The data collected were used to determine the percentage of water absorption using the following formula:

(1) $$\begin{array}{c} \%\;\mathrm{water}\text{ absorption}=\frac{\text{initial weight}-\text{final }\mathrm{weight}\;}{\text{initial weight}}\times 100. \end{array}$$

### 2.6. Examination of Fractured Surfaces

A scanning electron microscope was used to examine the surface morphology of the composites. Certain portion from each composition was cut, and surfaces were prepared. The surfaces were also coated with a gold Quorum coating machine (Q150RES) to make them conductive. The coated surfaces were examined by means of a PhenomProX scanning electron microscope (SEM) with an energy dispersive spectroscope (EDS), operated at 15 kV.

## 3. Results and Discussion

### 3.1. Mechanical Properties

#### 3.1.1. Ultimate Tensile Strength

**Figure 3:**
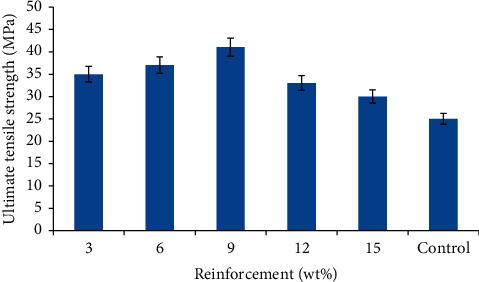
Variation in the ultimate tensile strength of the developed composites and the control.

Figure 3 shows the variation in the ultimate tensile strength for the snail shell-reinforced waste plastic composites. The ultimate tensile strength (UTS) of the recycled plastic-based composites was noticed to increase gradually with the addition of particulate snail shell from 3–9 wt% before decreasing. Despite this trend, developed composites have UTS higher than that of the control sample. The composite sample with the highest value was found at 9 wt% reinforcement with a value of 41 MPa culminating to 64% enhancement. The improvement in the tensile strengths of the reinforced waste plastic-based composites can be attributed to rigidity of the reinforcement and adequate interfacial adhesion between the two phases. This occurrence of improved tensile strength with respect to SSP is a function of an improved interfacial interaction between the surfaces of SSP and RWP which results in an efficient stress transfer mechanism within the composite system. Effective stress transfer in filled-polymer composites is the key strengthening mechanism for tensile strength enhancement. Also, the presence of calcium carbonate in the snail shell particle [17], which is hard and rigid, enhances the composite's resistance to deformation, thereby impeding dislocation motion. Another contributing factor was the chain interdiffusion and entanglement between the waste plastic and the snail shell particles which aids improvement in tensile strength in agreement with the research carried out by Daramola et al. [30].

The decrease in the ultimate tensile strength of samples with higher SSP compositions can be linked with an increase in the quantity of the particulate reinforcement in the RWP matrix. At high SSP addition, the strengthening mechanism becomes ineffective as observed with 12–15 wt% filled SSP. This occurred because the adhesion strength between the interfaces of RWP and SSP has been reduced. Owing to this fact, the stress transfer within the composite becomes ineffective, and this increases with an increasing filler content as the applied stress at this stage is principally bore by the RWP (the poorly bonded particles at this stage begin to debond from the RWP matrix upon the application of stress) resulting in a composite with decreased tensile strength. This can aid in the formation of agglomerates of SSP in the RWP matrix.

The ultimate tensile strength of polypropylene in the research carried out by Yanhui and Jinglong [31] is 23.108 MPa which is still lower than the optimum value recorded in this work showing that the reinforcing materials have played significant role in adding values to the recycled polypropylene.

#### 3.1.2. Young's Modulus of Elasticity

**Figure 4:**
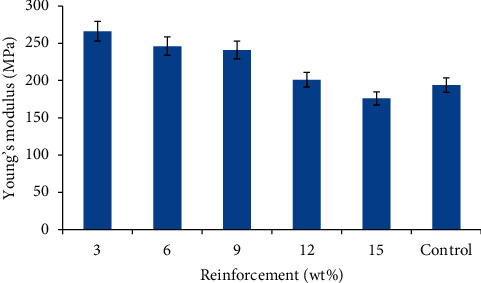
Variation in Young's modulus of the developed composites and the control.

Young's modulus is a measure of the stiffness of a material at the elastic region during tensile loading.  Figure 4 illustrates the Young's modulus of the snail shell particulate/recycled waste plastic composites and the control sample. There was a significant improvement in the Young's modulus of the composites produced within 3–9 wt%, though the modulus tends to decrease as the SSP content increases from 3–15 wt%. The composite produced with 3 wt% gave the optimum result with a value of 266 MPa while samples with 6 and 9 have 246 and 241 MPa, respectively. This shows that the presence of rigid and hard phase SSP in the RWP matrix within this range of SSP addition enhances the stiffness of the composites compared to the control sample with a value of 194 MPa. Also, the small particle size of the snail shell accounts for a high aspect ratio of the composite which enhances Young's modulus in agreement with the findings of [14]. The decrease in Young's modulus of the sample with 15 wt% SSP can be linked to poor dispersion of the particles in the RWP matrix and increase in the quantity of SSP in the matrix. This lowers the crosslinking density of the composites and aids in the detachment of the RWP from the hard phase SSP dispersed during loading. These also contribute to the reason why even within the range where the modulus was highly enhanced (3–9 wt%), it was observed that the modulus decreases as the snail shell particulate increases but was only highly pronounced at 15 wt% addition. From the sample with optimum modulus, the enhancement was 37% compared to the control sample.

The optimum Young's modulus is also higher that Young's modulus of polypropylene which is one of the high-performance plastics used in automobiles as revealed in the work of Yanhui and Jinglong [31] with a value of 176.79 MPa.

#### 3.1.3. Impact Strength

**Figure 5:**
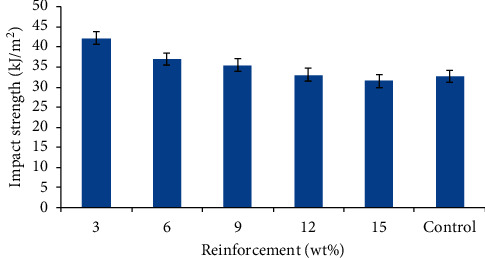
Variation in impact strength of the developed composites and the control.

The variation in the impact strength of the control and SSP/RWP composites was presented in Figure 5. There was an appreciable increase in the impact strength of the sample with 3 wt% of SSP with a value of 4.22 kJ/m^2^ after which the impact strengths tend to decrease. This shows that the addition of a small amount of the SSP content was the needed quantity to positively affect the impact strength of the RWP composites. It has been reported that the impact strength of filled polymers depends on the degree of interfacial bonding between the polymer matrix and the filler [16]. This reveals that the SSP bonds properly with the RWP matrix with a small addition of the snail shell particulate. Similarly, the presence of well-dispersed fillers helps to hinder crack propagation. The slight decrease in the impact strength of other compositions can be attributed to improper bonding resulting from an increased amount of the SSP which tends to promote agglomeration. The results obtained follow trends similar to those in Figure 4. However, it is noteworthy that proper adhesion of optimum SSP addition to RWP ameliorates the impact strength and gave 29% enhancement. From the mechanical test results, it was observed that these bio-based composite materials can be used as door sills in the interior of automobiles. The volume of SSP in polypropylene-based RWP should be within 3–9 wt% for an optimum value.

The impact strength of polypropylene at room temperature is about 22 kJ/m^2^ as recorded in the work of Yetistiren et al. [32], which is lower than that of any of the composites developed in this work.

### 3.2. Wear Index

**Figure 6:**
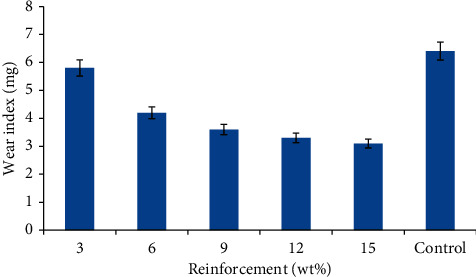
Wear index of the developed composites and the control.

Wear index indicates the rate at which materials deteriorate when in contact with a sliding/rotating surface. The lower the wear index, the more resistant the material to abrasion. The variation in the wear behaviour of the SSP/RWP composites and the control is illustrated in Figure 6. The wear index was observed to be decreasing as the SSP increases from 3 to 15 wt%, though a sharp decrease was observed from 3–6 wt% compared to others. The highest wear resistance was obtained at 15 wt% SSP addition with a value of 3.1 mg contrary to what was obtained for the control sample with a value of 6.4 mg, having the least wear resistance. This culminated to 52% enhancement in the abrasion resistance. This can be attributed to the resistance offered by the hard phase SSP to abrasion. Higher volume of the SSP supports the load bearing in this regard and, hence, prevents detachment of the reinforcement during abrasion loading influence. This finding was in agreement with previous submission where it was revealed that the addition of some selected agro waste aids the reduction in the abrasion effect on the developed composites [29]. These showed that the addition of biofillers into polymers can promote the reduction in the production of nondegradable materials in structural applications.

### 3.3. Water Absorption

**Figure 7:**
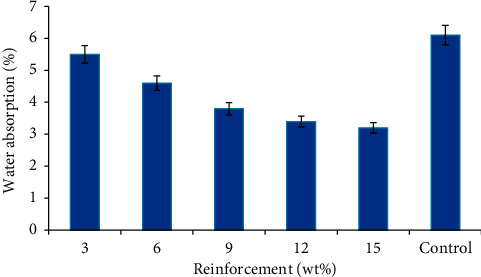
Variation in water absorption of the developed composites and the control.

The variation in the ability of the developed RWP composites to absorbed water is shown in Figure 7. A trend similar to that in Figures 5 and 6 was observed with the rate of water absorption for the composites decreasing as the SSP addition increases. The sample with 15 wt% SSP addition with a value of 3.2% gave the best water resistance potential. The water absorption property of PMCs reinforced with particulate fillers and their derivatives is dependent on the amount of the particle, dispersion efficiency, immersion temperature, area exposed to water, permeability of particulates, void content in the PMC, and hydrophilicity of the individual component. Previous research studies have shown that poor wettability and interfacial adhesion between fillers and polymer matrix can cause hydrophilicity of the composite [33–35]. In the case of the composites produced, the resistant to water absorption can be attributed to the rigid nature of the snail shell particulate and the filling of the pores and/or voids within the RWP matrix as the SSP content increases. The sample with the optimum resistance to water absorption gave 91% enhancement compared to the control sample. The presence of a significant amount of this hard and rigid CaCO_3_ phase from SSP in the RWP matrix has brought about a great improvement in the wear and water-repellant potentials of the developed composites. Water uptake reduces as the amount of SSP increases because most of the pores/voids are being filled by the SSP, thereby reducing the number of possible pores that water can fill. Unlike plant fibers/particles, some animal-based particles tend to be more hydrophobic. Hence, when used as reinforcement in polymer matrix, they tend to cause reduction in water/moisture intake different from plant-based reinforcement that will cause an increase in the water/moisture intake.

From wear and water absorption test results, it was observed that these bio-based composite materials can also be used as the floor panel in the interior of automobiles where good resistance to these environmental challenges will be encountered. The volume of SSP in polypropylene-based RWP should be 15 wt% for an optimum value.

### 3.4. Examination of Fractured Surfaces

**Figure 8:**
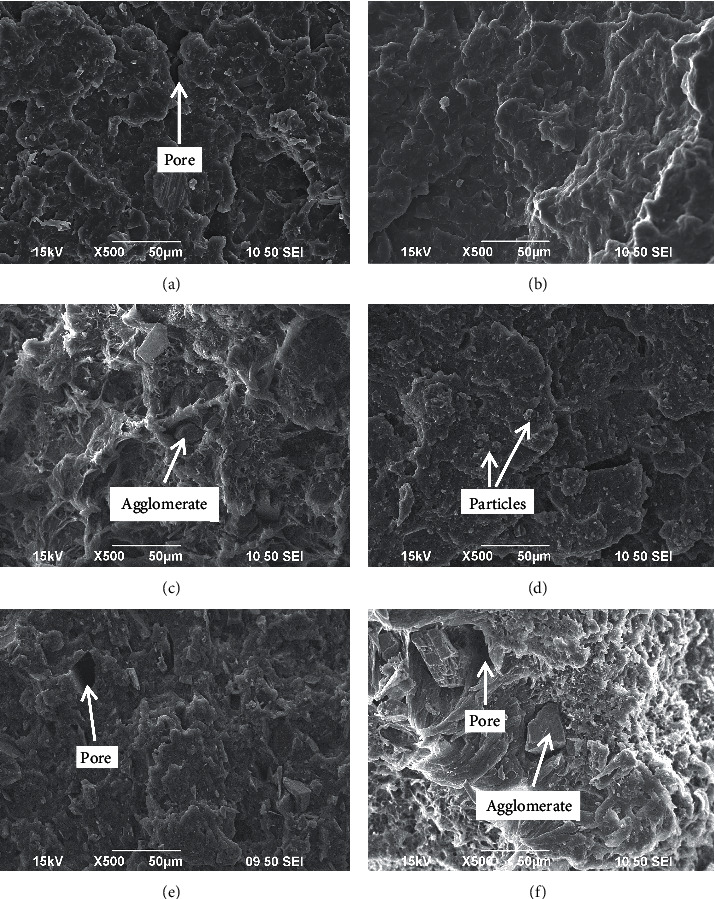
Scanning electron micrograph of the developed SSP/RWP composites and control. (a) Morphology of RWP, (b) morphology of 3 wt%, (c) morphology of 6 wt%, (d) morphology of 9 wt%, (e) morphology of 12 wt%, and (f) morphology of 15 wt%.

A detailed investigation of the filler morphology within the matrix shows the manner in which the SSP was dispersed in the RWP matrix. To have a clear understanding of the factors that can be responsible for the observed mechanical, physical, and wear properties of the composites developed, SEM images of the fractured surfaces were as shown in Figure 8.

Figure 8(a) shows the morphology of RWP in which some pores/voids were noticed, and this may likely be the reason for weak mechanical and wear properties observed when compared to the developed composites.  Figure 8(b) revealed the morphology of the sample with 3 wt% SSP addition where it was shown that the SSP are evenly dispersed within the RWP matrix without agglomeration. This potential contributed to the observed enhancement obtained from sample in response to Young's modulus of elasticity and impact strength properties. The morphology of the sample with 6 wt% SSP addition is shown in Figure 8(c). It was revealed from the image that the particulate was also properly blended with RWP but not as noticed in Figure 8(b). Few agglomerates were noticed with pores being generated around them. This feature was responsible for moderate improvement in the mechanical properties compared to other samples.  Figure 8(d) shows the micrograph of the sample with 9 wt% of SSP. The particles were well dispersed within the matrix. However, there are few cracks and pores which contribute to the observed response to mechanical properties examined. The composite has the best tensile strength which implies that the observed cracks and pores were not much pronounced to adversely affect the composite mechanical properties. The distribution of SSP with 12 wt% was as shown in Figure 8(e). Few agglomerates with some cavities/pores in certain portion of the micrograph were present, and these have an adverse effect on the mechanical properties. The SEM image of the sample with 15 wt%, as shown in Figure 8(f), reveals the presence of agglomeration and pores in a higher proportion compared to other samples. Hence, the mechanical properties were further depreciated. Nevertheless, a higher volume of SSP led to the appearance of a more hard and rigid phase in the RWP which contributed to the observed improved wear and water-repellant potentials in the developed composites.

## 4. Conclusions

Based on the outcome of the research into the possibility of using recycled waste plastics for interior automobile application by reinforcing with a biofiller for eco-friendly materials development, the following conclusions are derived from the investigation; hard and rigid phase snail shell particulate-derived CaCO_3_ that was added to the recycled polypropylene waste plastics led to the enhancement in mechanical, wear, and moisture-repellant properties of the developed bio-based composites. These improvements were possible because of adequate wetting and good interfacial adhesion at the SSP-RWP interface. Different volume of the SSP affect the properties the developed composites differently, while mechanical properties were improved within 3–9 wt%, and wear and water-repellant potentials were highly enhanced with the addition of 15 wt% of SSP. The work revealed the potentials in the use of bio-filler-recycled waste plastic-based composites for most automobile interiors where moderate mechanical and good wear and water resistance properties are desirable. It is envisaged that, after reuse, the developed polymer composite will degrade easily due to aging when disposed, but this issue may have to be investigated for authentication.

## Figures and Tables

**Table 1:** Composite composition.

Composition	RWP (wt%)	SSP (wt%)	Dioctyl phthalate (wt%)	Zinc stearate (wt%)
Control	98	0	0.5	1.5
3	95	3	0.5	1.5
6	92	6	0.5	1.5
9	89	9	0.5	1.5
12	86	12	0.5	1.5
15	83	15	0.5	1.5

## Data Availability

The data used to support the findings of this study are included within the article.

## References

[1] Ughale V. B., Singh K. K., Kumar P. (2016). Review paper on development of polymer composite material and its characterization. *IOSR Journal of Mechanical and Civil Engineering*.

[2] Prabhakar K., Debnath S., Ganesan R., Kumar P. (2018). A review of mechanical and tribological behavior of polymer composite materials. *IOP Conference Series. Material Science and Engineering*.

[3] Matabola K. P., De Vries A. R., Moolman F. S., Luyt A. S. (2009). Single polymer composites: a review. *Journal of Materials Science*.

[4] Kabir M. M., Wang H., Lau K. T., Cardona F., Aravinthan T. (2012). Mechanical properties of chemically-treated hemp fibre reinforced sandwich composites. *Composites Part B: Engineering*.

[5] Aziz S. H., Ansell M. P. (2004). The effect of alkalization and fibre alignment on the mechanical and thermal properties of kenaf and hemp bast fibre composites: part 1—polyester resin matrix. *Composites Science and Technology*.

[6] Cantero G., Arbelaiz A., Llano-Ponte R., Mondragon I. (2003). Effects of fibre treatment on wettability and mechanical behaviour of flax/polypropylene composites. *Composites Science and Technology*.

[7] Oladele I. O., Omotoyinbo J. A., Aiyemidejor S. H. (2014). Mechanical properties of chicken feather and cow hair fibre reinforced high density polyethylene composites. *International Journal of Science and Technology*.

[8] Oladele I. O., Okoro A. M., Omotoyinbo J. A., Khoathane M. C. (2018). Evaluation of the mechanical properties of chemically modified chicken feather fibres reinforced high density polyethylene composites. *Journal of Taibah University for Science*.

[9] Davis G., Song J. H. (2006). Biodegradable packaging based on raw materials from crops and their impact on waste management. *Industrial Crops and Products*.

[10] Miller R. (2005). *The Landscape for Biopolymers in Packaging: Miller-klein Associates Report Summary and Full Report Available from the National Non-food Crops Centre*.

[11] Grigore M. E. (2017). Methods of recycling, properties and applications of recycled thermoplastic polymers. *Recycling*.

[12] Agunsoye J. O., Aigbodion V. S. (2013). Bagasse filled recycled polyethylene bio-composites: morphological and mechanical properties study. *Results in Physics*.

[13] Vairavan M. (2013). Effect of fly ash filler size on mechanical properties of polymer matrix composites. *International Journal of Mining, Metallurgy, and Mechanical Engineering*.

[14] Fu S.-Y., Feng X.-Q., Lauke B., Mai Y.-W. (2008). Effects of particle size, particle/matrix interface adhesion and particle loading on mechanical properties of particulate-polymer composites. *Composites Part B: Engineering*.

[15] Oladele I. O., Olajide J. L., Amujede M. (2016). Wear resistance and mechanical behaviour of epoxy/mollusk shell biocomposites developed for structural applications. *Tribology in Industry*.

[16] Adeyanju B. B., Oladele I. O., Abosede O. (2017). Characterization of snail shell reinforced polyester composites. *International Journal of Research and Engineering*.

[17] Tepsila S., Suksri A. (2018). Silicone rubber insulator using organic filler from golden apple snail shells. *Journal of Fundamental and Applied Sciences*.

[18] Jose J., Jyotishkumar P., George S. M., Thomas S. (2011). *Recent Developments in Polymer Recycling*.

[19] Srivastava V., Srivastava R. (2013). Advances in automotive polymer applications and recycling. *International Journal of Innovative Research in Science, Engineering and Technology*.

[20] Kim J.-P., Yoon T.-H., Mun S.-P., Rhee J.-M., Lee J.-S. (2006). Wood-polyethylene composites using ethylene-vinyl alcohol copolymer as adhesion promoter. *Bioresource Technology*.

[21] Bismarck A., Baltazar-Y-Jimenez A., Sarikakis K. (2006). Green composites as panacea? socio-economic aspects of green materials. *Environment, Development and Sustainability*.

[22] Ashori A. (2008). Wood-plastic composites as promising green-composites for automotive industries!. *Bioresource Technology*.

[23] Ning H., Janowski G. M., Vaidya U. K., Husman G. (2007). Thermoplastic sandwich structure design and manufacturing for the body panel of mass transit vehicle. *Composite Structures*.

[24] Klaus F., Abdulhakim A. A. (2013). Manufacturing aspects of advanced polymer composites for automotive applications. *Applied Composite Materials*.

[25] Mohanty A. K., Misra M., Hinrichsen G. (2000). Biofibres, biodegradable polymers and biocomposites: an overview. *Macromolecular Materials and Engineering*.

[26] ASTM (2008). *American Society of Testing and Materials, Standard Test Method for Tensile Property of Polymer Matrix composites: D3039-08*.

[27] ASTM (2007). *American Society of Testing and Materials, Standard Test Method for Flexural Property of Polymer Matrix Composite Materials: D7264M-07*.

[28] ASTM (2010). *Standard Test Methods for Determining the Izod Pendulum Impact Resistance of Plastics: D256-10*.

[29] Oladele I. O., Ibrahim I. O., Adediran A. A., Akinwekomi A. D., Adetula Y. V., Olayanju T. M. A. (2020). Modified palm kernel shell fiber/particulate cassava peel hybrid reinforced epoxy composites. *Results in Materials*.

[30] Daramola O. O., Oladele I. O., Adewuyi B. O., Sadiku R., Agwuncha S. C. (2015). Influence of submicron agrowaste silica particles and vinyl acetate on mechanical properties of high density polyethylene matrix composites. *West Indian Journal of Engineering*.

[31] Yanhui L., Jinglong G. (2011). Mechanical properties and wear behaviour of polypropylene/carbon nanotube nanocomposites. *Advanced Materials Research*.

[32] Yetistiren H., Zeren A., Şahin T. (2012). Wear properties of glass fiber reinforced polypropylene rubberpoint. *KGK Rubberpoint*.

[33] Daramola O. O., Akinwekomi A. D., Adediran A. A., Akindote-White O., Sadiku E. R. (2019). Mechanical performance and water uptake behaviour of treated bamboo fibre-reinforced high-density polyethylene composites. *Heliyon*.

[34] Odusanya A. A., Bolasodun B., Madueke C. I. (2014). Property evaluation of hybrid seashell/snail shell filler reinforced unsaturated polyester composite in comparison with sea shell and snail shell filler reinforced unsaturated polyester composite. *The International Journal of Engineering and Science*.

[35] Samat N., Marini C. D., Maritho M. A., Sabaruddin F. A. (2013). Tensile and impact properties of polypropylene/microcrystalline cellulose treated with different coupling agents. *Composite Interfaces*.

